# Seasonal characteristics of nosocomial infection in a psychiatric hospital in China with different nosocomial prevention and control backgrounds: a retrospective study

**DOI:** 10.1038/s41598-024-65368-8

**Published:** 2024-07-24

**Authors:** Yufang Zhou, Shuili Chen, Youtian Wang, Jingyu Liang, Huaijie Li, Haishan Shi, Tianyang Miao, Shengwei Wu, Aixiang Xiao, Junrong Ye, Xing Zheng

**Affiliations:** 1 The Affiliated Brain Hospital, Guangzhou Medical University, No. 36, Mingxin Road, Liwan District, Guangzhou, 510370 China; 2 Key Laboratory of Neurogenetics and Channelopathies of Guangdong Province and the Ministry of Education of China, Guangzhou Medical University, Guangzhou, China; 3 Guangzhou University of Chinese Medicine, Guangzhou, China

**Keywords:** Nosocomial infection, Mental disorders, Psychiatry, Season, Infection diagnosis, Hospitalized patients, Neuroscience, Psychology, Diseases, Health care, Medical research, Risk factors, Signs and symptoms

## Abstract

This study aimed to investigate the relationship between various prevention and control measures for nosocomial infections (NIs) in psychiatric hospitals and patients with mental disorders. This study aimed to determine the characteristics of NIs in psychiatric hospitals and provide a reference for infection prevention and control in this setting. Data from the NI monitoring system of a psychiatric hospital in southeastern China were analysed. Patients who were hospitalized for mental disorders from January 1, 2016, to November 30, 2019, were classified into the non-COVID-19 containment group (NC19C group, *n* = 898), while those who were hospitalized from January 25, 2020, to November 30, 2022, were classified into the COVID-19 containment group (C19C group, *n* = 840). The data were analysed using SPSS version 22.0, and independent sample *t* tests, chi-square tests, correlation analyses, and multivariate logistic regression analyses were performed. A significance level of *P* < 0.0024 was applied. The incidence rate of NIs was higher in autumn in the NC19C group, while no seasonal difference was detected in the C19C group (*P* < 0.0024). Further analysis revealed that in the C19C group, the risk of hospitalized patients with mental disorders developing hospital-acquired pneumonia in spring was 0.362 times that in winter (*OR* = 0.362, 95% *CI* = 0.200 ~ 0.656, *P* = 0.001), and in summer, the risk was 0.342 times that in winter (*OR* = 0.342, 95% *CI* = 0.185 ~ 0.633 *P* = 0.001). Patients aged 18–44 years had a 4.260 times higher risk of developing hospital-acquired upper respiratory tract infections than did those aged 60 years and older (*OR* = 4.260, 95% *CI* = 2.143 ~ 8.470; *P* = 0.000). The risk of acquiring urinary tract infections in the hospital was 0.324 times greater among patients aged 18–44 years than for patients aged 60 years and older (*OR* = 0.324, 95% *CI* = 0.171–0.613; *P* = 0.001). The NC19C group did not exhibit the aforementioned differences. During the NC19C period, differences were observed in the diagnosis of hospital-acquired infections and sex (all *P* = 0.000). Psychiatric hospitals exhibit distinct nosocomial infection characteristics under the context of various infection control measures. Against the backdrop of strengthened infection control, the nosocomial infection characteristics of psychiatric hospitals may be associated with the features of mental disorders.

## Introduction

Nosocomial infections (NIs), also known as hospital-acquired infections (HAIs), occur during a patient’s hospital stay and do not include infections present upon admission or in the latent period. NIs include infections contracted within the hospital premises, post-discharge, and by hospital staff but excludes preexisting or infections that were incubating before admission^1^. According to the research findings, the incidence of hospital-acquired infections varies among different countries and regions: in the United States, it is 3.2%^2^; in a hospital in the Kerman Shah Province of Iran, it is 3.3%^3^; and in the European Union Economic Area, it is 6.5%^4^.

Common NIs include hospital-acquired pneumonia (HAP), hospital-acquired upper respiratory tract infections (HAURIs), hospital-acquired urinary tract infections (HAUTIs), hospital-acquired skin infections (HASIs), and others^5^. NIs are divided into exogenous infection and endogenous infection according to the infection route, among which the most common pathogenic bacteria include *Klebsiella pneumoniae*, *Escherichia coli*, *Proteus*, *P. aeruginosa*, and *Acinetobacter baumannii*. The pathogenic bacteria at different sites of NIs also differ^1,6^. The common NI varies across regions. In a hospital in Kermanshah Province, Iran, there were more men than women with nosocomial infections, and the most common NI was urinary tract infection (38.5%)^3^; however, in northern China, lower respiratory tract infections were the most common NI, with a higher incidence observed among females^7^. Developing countries report higher rates of surgical site infection than developed countries^8^. Pneumonia is the most common NI in the United States^2^. The most effective and simple way to control hospital infections is to control the source of infection, cut off transmission routes, and protect susceptible populations.

The global outbreak of the novel coronavirus pandemic in January 2020 prompted intensified hospital infection prevention and control measures. In Japan, hospitals enhanced existing protocols, mandating mask wearing, stringent disinfection, room isolation, and visitor restrictions^9^. In China, government guidelines further emphasized personnel training, joint prevention efforts, and daily supervision^10^.

Mental disorders encompass cognitive, emotional, and behavioural abnormalities and include schizophrenia, depression, and bipolar affective disorder, among others^11^. Schizophrenia is a type of mental disorder characterized by delusions, hallucinations, disordered thinking, and behavioural disturbances^12^. Depression is a type of mental disorder primarily characterized by a depressed mood or loss of pleasure and is often accompanied by cognitive, behavioural, or autonomic nervous system symptoms^12^. Bipolar disorder is a type of mental disorder that involves both manic and depressive episodes^11^. Compared with 1990, the prevalence of various mental disorders significantly increased in 2019, which has also led to an increase in the disability and burden caused by mental disorders^13^. Studies have shown a link between season and mental illness; for instance, winter birth increases schizophrenia risk in the Northern Hemisphere^14^. In recent years, the relationship between inflammatory mechanisms and mental disorders has been extensively studied. There is evidence that the levels of certain inflammatory factors are higher in patients with mental disorders than in healthy individuals^15,16^.

Similarities exist between mental disorders and nosocomial infections, including correlations between the environment and inflammation. However, research on nosocomial infections in psychiatric hospitals is limited.

This study is the first to compare nosocomial infections in psychiatric hospitals between the COVID-19 containment period and the non-COVID-19 containment period. By analysing the incidence of hospital-acquired infections in psychiatric hospitals with different infection prevention and control measures, we aimed to objectively understand the characteristics of hospital-acquired infections in psychiatric hospitals. The goal of this study is to provide targeted recommendations and strategies suitable for the actual situation of psychiatric hospitals. These findings should serve as a reference for strengthening infection prevention and management for administrators of psychiatric hospitals.

## Methods

This retrospective study examined patients with schizophrenia, depression, and bipolar disorder admitted to a psychiatric hospital in Southeast China. Patient data were obtained from the hospital’s infection surveillance system. Patients were divided into two groups according to the time of NI diagnosis: the non-COVID-19 containment group (NC19C, *n* = 898) was diagnosed with NI between January 2016 and November 2019, and the COVID-19 containment group (C19C, *n* = 840) was diagnosed with NI between January 25, 2020, and November 2022. The inclusion criteria were as follows: patients aged 18–100 years; Han nationality; diagnosed with schizophrenia, depression, or bipolar affective disorder according to the International Classification of Diseases, 10th Revision (ICD-10)^12^; hospitalized for more than one month; and no infectious diseases upon admission. The exclusion criteria were as follows: major medical abnormalities such as central nervous system diseases; acute, unstable, or life-threatening conditions (e.g., cancer); organic mental disorders; and alcohol or substance dependence.

The data collected from the infection monitoring system included patient sex, age, psychiatric diagnosis, and infection diagnosis. According to previous methods^17^, patients were categorized into three age groups: 18–44 years, 45–59 years, and 60 years and above. Infection diagnoses were based on infectious disease diagnoses reported by physicians, including HAP, HAURI, HAUTI, HASI, and other infections. Other infections included ophthalmic infections, gastrointestinal infections, soft tissue infections, tracheobronchitis, and asymptomatic bacteriuria, which were grouped due to their low incidence. HAP^5^ refers to pneumonia that is caused by pathogenic bacteria and results in pulmonary inflammation after 48 h of hospital admission, during which no infection or incubation period occurred. HAURI refers to acute inflammation of the nasal cavity, pharynx, and larynx acquired in the hospital setting. HAUTI refers to inflammatory reactions of the urinary tract epithelium acquired in the hospital setting, often accompanied by bacteriuria and pyuria, and characterized by clinical manifestations such as frequency, urgency, and dysuria.

During the NC19C period, standard precautions were implemented, including mask wearing by hospital staff and regular ventilation and disinfection of hospital wards according to the Regulations for Management of Hospital Environmental Surface Cleaning and Disinfection. However, no restrictions were imposed on patients, visiting relatives, or other visitors regarding mask wearing, activity range, or health status assessment. Visitors could enter the ward during daytime visiting hours, and patients and visitors were not required to wear masks or report their health status.

During the C19C period, the psychiatric hospital implemented the following enhanced infection control measures beyond societal standards. (1) Strict personnel management measures were implemented to control sources of infection and interrupt transmission routes, with all staff members (medical and nursing staff, attendants, cleaners, and security personnel) residing and working within the hospital premises, minimizing visits to crowded places, undergoing testing every 1–2 days during work hours, and undergoing health screenings twice daily. All staff members resided in the hospital premises during local epidemic periods. “Virtual (online) visits” were implemented for all hospitalized patients, and external personnel were prohibited from contacting patients. New patients were isolated in single rooms for 7 days. Hospital staff and patients reported their health status daily, and employees with suspected symptoms such as fever, fatigue, dry cough, sore throat, loss of smell (taste), or diarrhoea were not allowed to return to work, while patients with suspected symptoms were isolated in separate wards. Medical staff used personal protective equipment (PPE) in accordance with national guidelines, and dedicated personnel were responsible for daily supervision and the enforcement of infection control measures, thus ensuring a high level of prevention and control. (2) Noncontact mailing of items was implemented, with all life items undergoing unified surface disinfection and being stored for 3 days before being brought into the ward for use. The essential supplies were transferred by staff wearing protective equipment, disinfected at the hospital entrance, and brought back to the department for use after disinfection to reduce contact transmission pathways. (3) Vulnerable populations were protected by having hospitalized patients voluntarily wearing masks during daytime rehabilitation treatment or examinations, wearing surgical masks when leaving the ward for treatment or examinations, and receiving treatment or examinations in staggered time periods. No COVID-19 infections were reported among hospitalized patients or staff during the lockdown period, indicating the effectiveness of infection control measures.

Statistical analysis was performed using SPSS 22.0. Kolmogorov‒Smirnov tests were used to assess the normality of the distribution of variables. Continuous variables are presented as (*χ*^*2*^ ± *SD*) for normally distributed data. Independent sample *t* tests were used for between-group comparisons of continuous variables, and chi-square tests (*χ*^*2*^) were used for between-group comparisons of categorical variables. Multivariate logistic regression analysis was used to identify risk factors for infections, with a Bonferroni corrected *P* = 0.0024 considered to indicate statistical significance.

The specific steps of the statistical analysis were as follows. First, independent sample t tests and chi-square tests were used to compare the general characteristics, including sex, age, psychiatric diagnoses, infection diagnoses and seasonal distribution, between the containment and non-containment groups. Subsequently, chi-square tests were employed to compare the distributions of sex, age, psychiatric diagnosis, infection diagnosis, and whether there was epidemic containment across different seasons. The analysis also included evaluations of the distribution of sex, age, psychiatric diagnosis, and infection diagnosis across different seasons and epidemic containment periods. Next, collinearity diagnostics were performed, ensuring that the collinearity values were all below 2. Then, multiple logistic regression analysis was conducted. In the multiple logistic regression analysis, infection diagnosis was treated as the dependent variable; season, epidemic containment status, and their interaction term were included as independent variables; and age, sex, and psychiatric diagnosis were entered as covariates. A backwards stepwise method was used for automatic variable selection. Subsequently, stratified multiple logistic regression analysis was performed based on different epidemic containment periods. In the stratified multiple logistic regression analysis, infection diagnosis was the dependent variable, and season, epidemic containment status, age, and sex were included as independent variables.

## Results

Table 1 presents the general characteristics of the patients during the C19C and NC19C periods, including age, sex, psychiatric diagnosis, infection diagnosis and seasonal distribution. The results revealed that there was no significant difference in sex between psychiatric inpatients with hospital-acquired infections in the C19C group and those in the NC19C group (all *P* > 0.0024). In terms of age distribution, inpatients with mental illnesses aged 18–44 years in the C19C group were more likely to develop NIs than those in the NC191C group (49.2% vs. 39.0%). In contrast, among patients aged 45–59 years, the proportion of patients who developed NIs in the C19C group was lower than that in the NC19C group (21.4% vs. 35.2%), whereas the proportion of those aged > 60 years who developed NI was similar in the two groups (29.4% vs. 25.8%) (*P* < 0.0024). In terms of psychiatric diagnosis, schizophrenia was the most prevalent diagnosis in both groups, followed by bipolar affective disorder and depression, with statistically significant differences (all *P* < 0.0024). For infection diagnosis, compared to the NC19C group, psychiatric inpatients had in the C19C group had higher rates of hospital-acquired pneumonia (HAP) (55.5% vs. 22.6%), lower rates of hospital-acquired upper respiratory tract infections (HAURIs) (12.7% vs. 33.3%), higher rates of hospital-acquired urinary tract infections (HAUTIs) (8.3% vs. 5.1%), lower rates of hospital-acquired skin infections (HASIs) (8.1% vs. 14.4%), and lower rates of other infections (15.4% vs. 24.6%); all of these differences were statistically significant (*P* < 0.0024). Regarding seasonal differences, the number of nosocomial infections among psychiatric inpatients was similar in each season in the C19C group; in the NC19C group, nosocomial infections were more common in autumn (37.0%) and least common in spring (15.1%), with intermediate rates in summer and winter (23.5%, 24.4%) (*P* < 0.05).

**Table 1:** Distribution of general situation under different infection control measures.

Variables	C19C	NC19C	*χ*^*2*^*/t*	*P*
Sex				
Males	525(62.5%)	520(57.9%)	3.820	0.051
Females	315(37.5%)	378(42.1%		
Age	44.99 ± 19.314	48.01 ± 15.956	− 3.543	0.000*
18 ~ 44	413(49.2%)	350(39.0%)	41.072	0.000*
45 ~ 59	180(21.4%)	316(35.2%)		
≥ 60	247(29.4%)	232(25.8%)		
Psychiatric diagnosis				
Schizophrenia	498 (59.3%)	639 (71.2%)	45.020	0.000*
Depression	101 (12.0%)	38 (4.2%)		
Bipolar affective disorder	241 (28.7%)	221 (24.6%)		
Infection diagnosis				
HAP	466 (55.5%)	203 (22.6%)	240.559	0.000*
HAURI	107 (12.7%)	299 (33.3%)		
HAUTI	70 (8.3%)	46 (5.1%)		
HASI	68 (8.1%)	129 (14.4%)		
Other infections	129 (15.4%)	221 (24.6%)		
Season				
Spring	223 (26.5%)	136 (15.1%)	52.736	0.000*
Summer	202 (24.0%)	211 (23.5%)		
Autumn	199 (23.7%)	332 (37.0%)		
Winter	216 (25.7%)	219 (24.4%)		

NC19C = non-COVID-19 containmales t, C19C = COVID-19 containmales t. * means *P* < 0.0024, which means that the results of this item are statistically significant. HAP means hospital-acquired pneumonia, HAURI means hospital-acquired upper respiratory tract infections, HAUTI means hospital-acquired symptomatic urinary tract infections, HASI means hospital-acquired skin infections.

Table 2 shows the distribution of nosocomial infections (NIs) across different seasons. There were no seasonal differences in the distributions of age, sex, psychiatric diagnosis, or infection diagnosis (all *P* > 0.0024). However, there were seasonal differences in nosocomial infections during different hospital epidemic containment periods. Specifically, in the spring, the incidence of NIs was higher in the C19C group than in the NC19C group (62.1% vs. 37.9%), while in the autumn, the incidence of NIs was lower in the C19C group than in the NC19C group (37.5% vs. 62.5%). The incidence rates of NI in summer and winter were similar between the two periods, with the differences being statistically significant (*P* < 0.0024).

**Table 2:** Distribution of general situation in different seasons (*χ*^*2*^).

	Spring	Summer	Autumn	Winter	*χ2*	*P*
Sex						
Males	214 (59.6%)	245 (59.3%)	320 (60.3%)	266 (61.1%)	0.345	0.951
Females	145 (40.4%)	168 (40.7%)	211 (39.7%)	169 (38.9%)		
Age						
18 ~ 44	170 (47.4%)	172 (41.6%)	245 (46.1%)	176 (40.5%)	7.927	0.243
45 ~ 59	100 (27.9%)	121 (29.3%)	152 (28.6%)	123 (28.3%)		
≥ 60	89 (24.8%)	120 (29.1%)	134 (25.2%)	136 (31.3%)		
Psychiatric diagnosis						
Schizophrenia	226 (63.0%)	277 (67.1%)	346 (65.2%)	288 (66.2%)	4.463	0.614
Depression	26 (7.2%)	38 (9.2%)	43 (8.1%)	32 (7.4%)		
Bipolar affective disorder	107 (29.8%)	98 (23.7%)	142 (26.7%)	115 (26.4%)		
Infection diagnosis						
HAP	142 (39.6%)	137 (33.2%)	192 (36.2%)	198 (45.5%)	27.632	0.006
HAURI	84 (23.4%)	92 (22.3%)	134 (25.2%)	96 (22.1%)		
HAUTI	17 (4.7%)	33 (8.0%)	47 (8.9%)	19 (4.4%)		
HASI	38 (10.6%)	56 (13.6%)	60 (11.3%)	43 (9.9%)		
Other infections	78 (21.7%)	95 (23.0%)	98 (18.5%)	79 (18.2%)		
Whether the epidemic containment period						
C19C	223 (62.1%)	202 (48.9%)	199 (37.5%)	216 (49.7%)	57.736	0.000*
NC19C	136 (37.9%)	211 (51.1%)	332 (62.5%)	219 (50.3%)		

C19C means the COVID-19 containment, NC19C means the non-COVID-19 containment. HAP means hospital-acquired pneumonia, HAURI means hospital-acquired upper respiratory tract infections, HAUTI means hospital-acquired symptomatic urinary tract infections, HASI means hospital-acquired skin infections. * means *P* < 0.0024, which means that the results of this item are statistically significant.

Table 3 shows the distribution of NIs during different epidemic containment periods across various seasons. There were no seasonal differences in the distributions of sex (*P* > 0.0024). In the summer and autumn, psychiatric inpatients aged 18–44 years in the C19C group were more likely to develop NI than those in the NC19C group were (52.7% vs. 31.8%, 59.3% vs. 38.3%). Conversely, psychiatric inpatients aged 45–59 years in the C19C group were less likely to develop NI than were those aged 45–59 years in the NC19C group (20.5% vs. 37.4%, 12.6% vs. 38.3%). The likelihood of NI occurrence was similar for patients aged 60 and above in both groups (26.8% vs. 30.8%, 28.1% vs. 23.4%), with these differences being statistically significant (*P* < 0.0024). Differences in spring and winter were not statistically significant (*P* > 0.0024). Regardless of the season, psychiatric inpatients in the C19C group had the highest incidence of hospital-acquired pneumonia (HAP), whereas psychiatric inpatients in the NC19C group had the highest incidence of hospital-acquired upper respiratory tract infection (HAURI). These differences were statistically significant (*P* < 0.0024).

**Table 3:** Distribution of general situation under different infection control measures in different seasons (*χ*^*2*^).

	Spring			Summer			Autumn			Winter		
	C19C	NC19C	*P*	C19C	NC19C	*P*	C19C	NC19C	*P*	C19C	NC19C	*P*
Sex						H16						
Males	137 (61.4%)	77 (56.6%)	0.367	117 (57.1%)	130 (61.6%)	0.346	128 (64.3%)	192 (57.8%)	0.139	145 (67.1%)	121 (55.3%)	0.011
Females	86 (38.6%)	59 (43.4%)		88 (42.9%)	81 (38.4%)		71 (35.7%)	140 (42.2%)		71 (32.9%)	98 (44.7%)	
Age												
18 ~ 44	104 (46.6%)	66 (48.5%)	0.011	108 (52.7%)	67 (31.8%)	0.000*	118 (59.3%)	127 (38.3%)	0.000*	86 (39.8%)	90 (41.1%)	0.878
45 ~ 59	53 (23.8%)	47 (34.6%)		42 (20.5%)	79 (37.4%)		25 (12.6%)	127 (38.3%)		60 (27.8%)	63 (28.8%)	
≥ 60	66 (29.6%)	23 (16.9%)		55 (26.8%)	65 (30.8%)		56 (28.1%)	78 (23.4%)		70 (32.4%)	66 (30.1%)	
Psychiatric diagnosis												
Schizophrenia	142 (63.7%)	84 (61.8%)	0.338	114 (55.6%)	163 (77.3%)	0.000*	110 (55.3%)	236 (71.1%)	0.000*	132 (61.1%)	156 (71.2%)	0.028
Depression	19 (8.5%)	7 (5.1%)		35 (17.1%)	3 (1.4%)		25 (12.6%)	18 (5.4%)		22 (10.2%)	10 (4.6%)	
Bipolar affective disorder	62 (27.8%)	45 (33.1%)		56 (27.3%)	45 (21.3%)		64 (32.2%)	78 (23.5%)		62 (28.7%)	53 (24.2%)	
Infection diagnosis												
HAP	112 (50.2%)	30 (22.1%)	0.000*	107 (52.2%)	33 (15.6%)	0.000*	112 (56.3%)	80 (24.1%)	0.000*	138 (63.9%)	60 (27.4%)	0.000*
HAURI	33 (14.8%)	51 (37.5%)		23 (11.2%)	69 (32.7%)		22 (11.1%)	112 (33.7%)		29 (13.4%)	67 (30.6%)	
HAUTI	15 (6.7%)	2 (1.5%)		20 (9.8%)	13 (6.2%)		24 (12.1%)	23 (6.9%)		11 (5.1%)	8 (3.7%)	
HASI	21 (9.4%)	17 (12.5%)		19 (9.3%)	37 (17.5%)		12 (6.0%)	48 (14.5%)		16 (7.4%)	27 (12.3%)	
Other infections	42 (18.8%)	36 (26.5%)		36 (17.6%)	59 (28.0%)		29 (14.6%)	69 (20.8%)		22 (10.2%)	57 (26.0%)	

C19C means the COVID-19 containment, NC19C means the non-COVID-19 containment. HAP means hospital-acquired pneumonia, HAURI means hospital-acquired upper respiratory tract infections, HAUTI means hospital-acquired symptomatic urinary tract infections, HASI means hospital-acquired skin infections. * means *P* < 0.0024, which means that the results of this item are statistically significant.

The results of the multivariate logistic regression in Table 4 indicate that the interaction terms between season and epidemic control period did not enter the equation, suggesting that there was no interaction effect between season and epidemic control period on hospital-acquired infection (HAI) diagnoses. Compared with other infections, sex, age, season, and different hospital infection control measures were significant risk factors for hospital-acquired pneumonia (HAP) (all *P* < 0.0024); sex, age, and different hospital infection control measures were significant risk factors for hospital-acquired upper respiratory tract infections (HAURIs) and hospital-acquired urinary tract infections (HAUTIs) (all *P* < 0.0024); and sex was a significant risk factor for hospital-acquired skin infections (HASIs) (*P* < 0.0024). Further stratified analysis results are presented in Tables 5 and 6.

**Table 4:** Multiple logistic regression results of different infection diagnosis.

Infection diagnosis	Independent variable	*B*	*P*	*Exp(B)*	95% *EXP(B) CI*
HAP	Sex				
	Males	1.420	0.000*	4.136	2.389 ~ 7.159
	Females	0.964	0.000*	2.621	1.502 ~ 4.573
	Age	− 0.017	0.000*	0.983	0.975 ~ 0.991
	Psychiatric diagnosis (Bipolar affective disorder)				
	Schizophrenia	− 0.267	0.119	0.766	0.548 ~ 1.071
	Depression	0.272	0.386	1.312	0.710 ~ 2.426
	Season (Winter)				
	Spring	− 0.596	0.004	0.551	0.369 ~ 0.823
	Summer	− 0.644	0.001*	0.525	0.356 ~ 0.775
	Autumn	− 0.171	0.373	0.843	0.578 ~ 1.228
	Whether the epidemic containment period (NC19C)				
	C19C	1.318	0.000*	3.736	2.806 ~ 4.975
HAURIs	Sex				
	Males	1.981	0.000*	7.249	4.043 ~ 12.998
	Females	1.717	0.000*	5.567	3.066 ~ 10.108
	Age	− 0.028	0.000*	0.973	0.963 ~ 0.982
	Psychiatric diagnosis (Bipolar affective disorder)				
	Schizophrenia	− 0.212	0.244	0.809	0.565 ~ 1.156
	Depression	0.086	0.806	1.090	0.548 ~ 2.169
	Season (Winter)				
	Spring	− 0.155	0.492	0.856	0.550 ~ 1.333
	Summer	− 0.253	0.239	0.776	0.509 ~ 1.183
	Autumn	− 0.018	0.929	0.982	0.655 ~ 1.472
	Whether the epidemic containment period (NC19C)				
	C19C	− 0.607	0.000*	0.545	0.393 ~ 0.755
HAUTIs	Sex				
	Males	− 4.534	0.000*	0.011	0.004 ~ 0.032
	Females	− 3.464	0.000*	0.031	0.011 ~ 0.088
	Age	0.042	0.000*	1.043	1.028 ~ 1.058
	Psychiatric diagnosis (Bipolar affective disorder)				
	Schizophrenia	− 0.786	0.004	0.455	0.267 ~ 0.776
	Depression	0.394	0.359	1.483	0.639 ~ 3.441
	Season (Winter)				
	Spring	− 0.264	0.495	0.768	0.360 ~ 1.639
	Summer	0.449	0.188	1.566	0.804 ~ 3.052
	Autumn	0.928	0.004	2.530	1.337 ~ 4.786
	Whether the epidemic containment period (NC19C)				
	C19C	1.175	0.000*	3.239	2.018 ~ 5.196
HASIs	Sex				
	Males	− 0.884	0.024	0.413	0.192 ~ 0.888
	Females	− 1.780	0.000*	0.169	0.076 ~ 0.375
	Age	0.013	0.023	1.013	1.002 ~ 1.025
	Psychiatric diagnosis (Bipolar affective disorder)				
	Schizophrenia	− 0.070	0.763	0.932	0.592 ~ 1.470
	Depression	− 0.307	0.554	0.736	0.266 ~ 2.035
	Season (Winter)				
	Spring	− 0.042	0.880	0.959	0.555 ~ 1.657
	Summer	0.077	0.765	1.080	0.653 ~ 1.785
	Autumn	0.150	0.557	1.161	0.705 ~ 1.913
	Whether the epidemic containment period (NC19C)				
	C19C	− 0.110	0.571	0.896	0.612 ~ 1.312

Infection diagnosis was the dependent variable. C19C means the COVID-19 containment, NC19C means the non-COVID-19 containment. HAP means hospital-acquired pneumonia, HAURI means hospital-acquired upper respiratory tract infections, HAUTI means hospital-acquired symptomatic urinary tract infections, HASI means hospital-acquired skin infections. * means *P* < 0.0024, which means that the results of this item are statistically significant.

**Table 5:** Influencing factors related to infection diagnosis in the C19C group (2020 ~ 2022 year).

Infection diagnosis	Independent variable	*B*	*P*	*Exp(B)*	95% *EXP(B) CI*
HAP	Sex (females)				
	Males	0.265	0.222	1.303	0.852 ~ 1.994
	Age (≥ 60)				
	18 ~ 44	1.484	0.000*	4.410	2.628 ~ 7.401
	45 ~ 59	0.038	0.885	1.039	0.619 ~ 1.743
	Psychiatric diagnosis (Bipolar affective disorder)				
	Schizophrenia	− 0.223	0.379	0.800	0.486 ~ 1.316
	Depression	0.321	0.451	1.379	0.598 ~ 3.181
	Season (Winter)				
	Spring	− 1.015	0.001*	0.362	0.200 ~ 0.656
	Summer	− 1.073	0.001*	0.342	0.185 ~ 0.633
	Autumn	− 0.902	0.006	0.406	0.214 ~ 0.769
HAURI	Sex (females)				
	Males	0.030	0.912	1.031	0.601 ~ 1.769
	Age (≥ 60)				
	18 ~ 44	1.449	0.000*	4.260	2.143 ~ 8.470
	45 ~ 59	0.247	0.502	1.281	0.622 ~ 2.636
	Psychiatric diagnosis (Bipolar affective disorder)				
	Schizophrenia	− 0.143	0.654	0.867	0.464 ~ 1.619
	Depression	0.009	0.986	1.009	0.353 ~ 2.890
	Season (Winter)				
	Spring	− 0.677	0.07	0.508	0.244 ~ 1.056
	Summer	− 0.994	0.013	0.370	0.169 ~ 0.810
	Autumn	− 0.914	0.027	0.401	0.178 ~ 0.903
HAUTI	Sex (females)				
	Males	− 1.128	0.001*	0.324	0.171 ~ 0.613
	Age (≥ 60)				
	18 ~ 44	− 2.150	0.000*	0.116	0.045 ~ 0.302
	45 ~ 59	− 1.137	0.004	0.321	0.149 ~ 0.690
	Psychiatric diagnosis (Bipolar affective disorder)				
	Schizophrenia	− 0.688	0.080	0.502	0.232 ~ 1.086
	Depression	0.733	0.187	2.081	0.700 ~ 6.185
	Season (Winter)				
	Spring	− 0.357	0.471	0.700	0.265 ~ 1.847
	Summer	0.131	0.789	1.140	0.438 ~ 2.967
	Autumn	0.615	0.205	1.850	0.715 ~ 4.788
HASI	Sex (females)				
	Males	0.872	0.014	2.392	1.194 ~ 4.791
	Age (≥ 60)				
	18 ~ 44	− 0.094	0.808	0.910	0.426 ~ 1.944
	45 ~ 59	− 0.531	0.149	0.588	0.286 ~ 1.210
	Psychiatric diagnosis (Bipolar affective disorder)				
	Schizophrenia	0.820	0.067	2.271	0.944 ~ 5.462
	Depression	0.571	0.433	1.770	0.425 ~ 7.378
	Season (Winter)				
	Spring	− 0.340	0.428	0.712	0.307 ~ 1.650
	Summer	− 0.289	0.512	0.749	0.315 ~ 1.780
	Autumn	− 0.609	0.209	0.544	0.210 ~ 1.407

Infection diagnosis was the dependent variable. C19C means the COVID-19 containment, NC19C means the non-COVID-19 containment. HAP means hospital-acquired pneumonia, HAURI means hospital-acquired upper respiratory tract infections, HAUTI means hospital-acquired symptomatic urinary tract infections, HASI means hospital-acquired skin infections. * means *P* < 0.0024, which means that the results of this item are statistically significant.

**Table 6:** Influencing factors related to infection diagnosis in the NC19C group (2016 ~ 2019 year).

Infection diagnosis	Independent variable	*B*	*P*	*Exp(B)*	95% *EXP(B) CI*
HAP	Sex (females)				
	Males	0.879	0.000*	2.409	1.600 ~ 3.626
	Age (≥ 60)				
	18 ~ 44	− 0.566	0.030	0.568	0.340 ~ 0.948
	45 ~ 59	− 0.309	0.207	0.734	0.454 ~ 1.187
	Psychiatric diagnosis (Bipolar affective disorder)				
	Schizophrenia	− 0.215	0.395	0.807	0.492 ~ 1.324
	Depression	0.137	0.798	1.147	0.400 ~ 3.291
	Season (Winter)				
	Spring	− 0.213	0.498	0.808	0.436 ~ 1.497
	Summer	− 0.723	0.013	0.485	0.274 ~ 0.858
	Autumn	0.084	0.738	1.088	0.664 ~ 1.781
HAURI	Sex (females)				
	Males	0.399	0.033	1.490	1.033 ~ 2.151
	Age (≥ 60)				
	18 ~ 44	0.779	0.002*	2.180	1.336 ~ 3.558
	45 ~ 59	0.32	0.196	1.377	0.848 ~ 2.236
	Psychiatric diagnosis (Bipolar affective disorder)				
	Schizophrenia	− 0.361	0.104	0.697	0.451 ~ 1.077
	Depression	0.072	0.878	1.075	0.425 ~ 2.717
	Season (Winter)				
	Spring	0.067	0.816	1.070	0.607 ~ 1.884
	Summer	0.046	0.858	1.047	0.631 ~ 1.739
	Autumn	0.315	0.190	1.371	0.855 ~ 2.197
HAUTI	Sex (females)				
	Males	− 1.344	0.001*	0.261	0.118 ~ 0.575
	Age (≥ 60)				
	18 ~ 44	0.102	0.830	1.107	0.437 ~ 2.804
	45 ~ 59	0.464	0.259	1.590	0.710 ~ 3.560
	Psychiatric diagnosis (Bipolar affective disorder)				
	Schizophrenia	− 0.596	0.120	0.551	0.260 ~ 1.168
	Depression	− 0.297	0.730	0.743	0.138 ~ 4.012
	Season (Winter)				
	Spring	− 0.598	0.404	0.550	0.135 ~ 2.244
	Summer	0.577	0.243	1.78	0.676 ~ 4.686
	Autumn	0.878	0.053	2.407	0.990 ~ 5.851
HASI	Sex (females)				
	Males	0.990	0.000*	2.690	1.676 ~ 4.318
	Age (≥ 60)				
	18 ~ 44	− 0.572	0.055	0.565	0.315 ~ 1.012
	45 ~ 59	− 0.285	0.302	0.752	0.437 ~ 1.292
	Psychiatric diagnosis (Bipolar affective disorder)				
	Schizophrenia	− 0.396	0.156	0.673	0.389 ~ 1.164
	Depression	− 0.993	0.232	0.370	0.073 ~ 1.887
	Season (Winter)				
	Spring	0.013	0.972	1.014	0.479 ~ 2.144
	Summer	0.175	0.584	1.192	0.637 ~ 2.231
	Autumn	0.379	0.214	1.461	0.804 ~ 2.653

Infection diagnosis was the dependent variable. C19C means the COVID-19 containment, NC19C means the non-COVID-19 containment. HAP means hospital-acquired pneumonia, HAURI means hospital-acquired upper respiratory tract infections, HAUTI means hospital-acquired symptomatic urinary tract infections, HASI means hospital-acquired skin infections. * means *P* < 0.0024, which means that the results of this item are statistically significant.

Table 5 presents the results of the multivariable logistic regression analysis of infection diagnosis in the C19C group. Compared to patients with other less common infections, patients aged 18–44 years were 4.410 times more likely to develop HAP than were those aged 60 years and above (*OR* = 4.410, 95% *CI* = 2.628 ~ 7.401; *P* = 0.000). The probability of HAP occurrence was 0.362 times lower in spring than in winter (*OR* = 0.362, 95% *CI* = 0.200 ~ 0.656, *P* = 0.001) and 0.342 times lower in summer than in winter (*OR* = 0.342, 95% *CI* = 0.185 ~ 0.633 *P* = 0.001). Compared to patients with other less common infections, patients aged 18–44 years were 4.260 times more likely to develop HAURIs than were those aged 60 years and older (*OR* = 4.260, 95% *CI* = 2.143 ~ 8.470; *P* = 0.000). Males were 0.324 times more likely to develop symptomatic HAUTIs than women were (*OR* = 0.324, 95% *CI* = 0.171–0.613; *P* = 0.001). Patients aged 18–44 years were 0.116 times less likely to develop symptomatic HAUTIs than were those aged 60 years and above (*OR* = 0.116, 95% *CI* = 0.045–0.302; *P* = 0.000).

Table 6 presents the results of the multivariable logistic regression analysis of infection diagnosis in the NC19C group. The probability of HAP occurrence in males was 2.409 times higher than that in females (*OR* = 2.409, 95% *CI* = 1.600–3.626;* P* = 0.000). The incidence of HAURIs in patients aged 18–44 years was 2.180 times higher than that in patients aged 60 years or older (*OR* = 2.180, 95% *CI* = 1.336 to 3.558; *P* = 0.002). The risk of symptomatic HAUTI occurrence in males was 0.61 times lower than that in females (*OR* = 0.261, 95% *CI* = 0.118–0.575; *P* = 0.001). The risk of HASI occurrence in males was 2.690 times higher than that in females (*OR* = 2.690, 95% *CI* = 1.676–4.318; *P* = 0.000).

## Discussion

To our knowledge, this is the first retrospective study to investigate the correlation between containment measures and NIs in patients with mental illness in China during the NC19C period and C19C period. We found the following results. (1) There was no seasonal variation in the incidence of NIs during the C19C period, but there was a seasonal difference in the incidence of HAP. During the NC19C period, while there were seasonal variations in the occurrence of NI, there was no seasonal difference in their diagnosis. (2) Most NIs exhibited age-related differences during the C19C period. In contrast, gender differences were more prominent during the NC19C period.

Previous research has indicated that the incidence of NIs in China varies by season. For instance, a hospital in Beijing reported that the prevalence rate of NIs was 2.4% in the second quarter of 2018^18^, while a hospital in Southwest China reported a rate of 3.27% in the second quarter of 2021^19^. The second and third quarters had the highest frequency of NIs in a hospital in southeast China, while the fourth quarter had the highest frequency of NIs in a hospital in northeast China^20^. However, there was no significant seasonal difference in NIs observed in a hospital in central China^21^. The data for this study were collected from a hospital located in Southeast China. Notably, during the NC19C period, this particular hospital experienced peak incidences of NI during the middle of autumn and summer, which aligns with findings from another hospital located in Southeast China, as reported by Xiao Xiong^20^. Due to significant regional environmental variations in China, seasonal differences in NI are primarily attributed to regional and environmental disparities. Our comparison of the results with previous findings appears to support this perspective. However, compared with the results of the lockdown period, we found that there was no significant seasonal difference in the occurrence of NI during the lockdown period when prevention and control were strengthened. These findings suggest that seasonal differences in NI may be linked to the effectiveness of prevention and control measures.

Second, during the lockdown period, when measures for preventing and controlling NI were strengthened, the incidence of HAP was highest in winter, although NI did not show seasonal differences. This phenomenon may be attributed to the characteristics of mental illness itself. Research has indicated that the onset season of schizophrenia exhibits two peaks, namely, the late cold season and late warm season^22^. The relationships between seasons and conditions such as depression, suicide, and mania were recognized by researchers as early as the last century^23^. For instance, in 1984, Norman E. Rosenthal et al.^24^ reported that seasonal affective disorder (SAD) is a disorder characterized by recurrent episodes of depression every year. Their study revealed that SAD patients tended to experience depression in the fall and winter months but showed improvement in their symptoms during the spring or summer months of the following year. Subsequent studies have also yielded similar results; for example, a study involving Alaskan adults revealed that 18.68% of adults met the criteria for winter depression, with a higher prevalence among women than men^25^. The cause of seasonal depression may be linked to seasonal changes in Gaba energy levels, glutaminergic system activity, melatonin (MT), and HPA axis hormones^15,26^.

These findings suggest that mental disorders exhibit seasonal patterns, with winter being a season associated with schizophrenia and depression. Several studies have indicated that the use of antipsychotic drugs, benzodiazepines, and other medications is linked to an increased incidence of HAP^27,28^. Chan et al.^29^ identified comorbidities, prolonged use of antipsychotics, severe psychiatric symptoms, and poor overall function as factors associated with HAP. In recent years, there has been extensive research on the relationship between inflammatory mechanisms and mental disorders. Furthermore, studies have demonstrated elevated concentrations of various inflammatory factors, such as interleukin, IL-1 receptor antagonists, soluble interleukin-2 receptor, IL-6, IL-8, IL-10, and tumour necrosis factor (TNF-α), in patients with schizophrenia compared to healthy individuals^15^. Similarly, in patients with depression, there was an increase in the concentrations of IL-8 and TNF-α^16^. Inflammatory stimulation may impact multiple brain circuits, including those involved in reward processing, motor function, threat detection, anxiety regulation, and emotional processing interoceptive awareness^20^. Additionally, inflammatory stimulation can affect structural and functional connectivity within the brain, which further influences psychiatric disorders^20^. Therefore, we hypothesize that the increased likelihood of HAP in patients with mental disorders during the winter, following the implementation of prevention and control measures for hospital-associated infections during the C19C period, may be attributed to several factors. First, it is possible that the exacerbation of mental illness leads to increased use of antipsychotic drugs and benzodiazepines, consequently increasing the incidence of drug-related HAP. Second, the exacerbation of mental illness may contribute to elevated levels of inflammatory factors within the body and a compromised functional state, potentially amplifying the risk of HAP when combined with psychiatric medication. However, during the non-COVID-19 lockdown period, there were no differences in NIs related to infectious diseases. This could be attributed to the increase in pathogenic sources due to unrestricted visitation and standard hospital hygiene measures, thus potentially masking the occurrence of HAP in psychosis patients.

During the C19C period, it was observed that individuals aged 18–44 years with mental illness were more susceptible to HAP and HAURI, while symptomatic HAUTI was more prevalent among elderly individuals over 60 years with mental illness. The onset peak of schizophrenia occurs between the ages of 10 to 25, 25 to 35, and after 60 years of age^30^, whereas depression typically peaks between the ages of 20 to 35^11^. In this study, the ages of psychiatric patients aged 18 to 44 years included the ages of onset of schizophrenia and depression, and the incidence of respiratory diseases in psychiatric patients was higher than that in the general population^31^. A study indicated that the prevalence of urinary tract infections increases with age, particularly among individuals over 65 years old. Furthermore, older adults are at a higher risk of developing urinary tract infections, and there is a gradual reduction in sex differences^32,33^. Additionally, individuals over 60 years old represent the most common age group for senile schizophrenia and senile depression. Patients with depression and anxiety often exhibit symptoms of ‘bladder somatic symptom disorder (SSD)’, such as frequent urination, urgency of urination, dysuria, and other related symptoms^34^. Treatment for depression and anxiety can help alleviate urinary symptoms. Notably, frequent urination, urgent urination, and dysuria are also key symptoms of urinary tract infection. In psychiatric hospitals, symptomatic urinary tract infections in some elderly patients with mental illness may be caused by the exacerbation of their depression and anxiety. Given that mental illness itself leads to elevated inflammatory factor levels^16^, psychiatrists should focus on identifying symptomatic urinary tract infections in elderly patients with mental illness resulting from an infection. It is important to consider whether there is an increase in mental illness or whether both factors contribute to this phenomenon. If the infection is caused by a urinary tract infection, the focus of treatment should be on eliminating the infection. If this is due to the exacerbation of mental illness, treatments should concentrate on improving patients’ depression and anxiety. If the occurrence of the disease is related to both infection and depression and anxiety, treatment should prioritize improving the patient’s depression and anxiety state while also reducing inflammation.

Finally, there were sex differences in HAP, HAUTI and HASI in patients with mental illness during the NC19C period, consistent with previous studies. For instance, Lopez-de-Andres et al.^35^ reported that men are more likely to develop HAP, while Chen et al.^36^ noted that men are more susceptible to infectious skin diseases. It is widely known that women are more likely to suffer from urinary tract infections than men^37^.

This study has strengths. This is the first study to analyse the characteristics of hospital atmosphere in psychiatric hospitals under different backgrounds of hospital prevention and control measures. However, this study has several limitations. First, it lacked patient infection strain data. Second, potential data bias in the samples cannot be ruled out.

## Conclusion

In summary, the characteristics of hospital-acquired infections in psychiatric patients vary under different backgrounds of hospital prevention and control measures. (1) Against the background of strengthening the prevention and control of hospital infection, there was no significant seasonal difference in the incidence of NIs among psychiatric inpatients in Southeast China. However, the incidence of hospital-acquired pneumonia is significantly higher during winter than in other seasons and significantly higher than the incidence of other NIs. Additionally, age differences existed in HAP, HAURI, and HAUTI during this period. It is important for psychiatrists to determine whether these differences are due to infection or exacerbation of mental illness. (2) In normal times, the incidence of nosocomial infection was highest in autumn, but there was no significant difference in the occurrence of nosocomial infection across seasons. Additionally, the diagnosis of NI in this group of inpatients during this period varied between the sexes, which aligns with findings from previous studies.

Our findings suggest that, in addition to focusing on infections in psychiatric hospitals, psychiatrists may also need to pay attention to the exacerbation of psychiatric symptoms and determine whether a patient’s infection is due to an NI, an exacerbation of mental illness, or both.

## Data Availability

The datasets used or analysed during the current study are available from the corresponding author upon reasonable request.
